# Structure–Activity Relationship Models to Predict Properties of the Dielectric Fluids for Transformer Insulation System

**DOI:** 10.3390/ijms25126654

**Published:** 2024-06-17

**Authors:** Mi Zhang, Hua Hou, Baoshan Wang

**Affiliations:** College of Chemistry and Molecular Sciences, Wuhan University, Wuhan 430072, China; zhangmi@whu.edu.cn (M.Z.); houhua@whu.edu.cn (H.H.)

**Keywords:** dielectric liquids, insulating oil, structure–activity relationship, electrostatic potential, group additivity

## Abstract

Mineral oils and synthetic and natural esters are the predominant insulating liquids in electrical equipment. Structure–activity relationship models to predict the key properties of pure insulating liquids, including pulse breakdown strengths, AC breakdown voltages, dielectric constants, flash points, and kinematic viscosities, have been proposed for the first time. Dependence of the specific properties on the molecular structures has been illustrated quantitatively in terms of surface area, statistical total variance, and average deviation of positive and negative electrostatic potentials, as augmented by molecular weight, volume, and ovality. Moreover, the individual contribution of the functional groups to viscosity has been revealed by an additive approach. The predicted properties are in good agreement with the experimental data. The present theoretical work provides new insights on the development of novel dielectric fluids.

## 1. Introduction

Petroleum-based mineral oils have been used for insulation and cooling purposes in power transformers for decades [1,2]. However, mineral oils present high health and environmental hazards due to poor neutral degradation and fire risk due to low flash points [3]. Recently, biodegradable and environmentally friendly natural esters derived from plants and seeds have attracted great interest for use as dielectric liquids with high flash points [4,5,6]. Nevertheless, natural esters always involve high viscosity and a high pour point, inferior oxidative stability, and low lightning breakdown voltage. The presence of β-H and C=C bonds in the molecular structures of natural esters accounts for such deteriorations [7,8,9]. Therefore, various synthetic esters have been obtained via chemical modification, namely, esterification or trans-esterification, to reduce viscosity and to improve thermal characteristics [10,11]. In view of the complexity of fatty acids in natural esters, it is challenging to use esterification as a tool to simultaneously balance both physicochemical and electrical properties.

Theoretically, the structure–activity relationship (SAR) models to predict viscosity, density, boiling and melting points, thermal conductivity, etc., were developed for many pure hydrocarbons [12]. In addition, many SAR models for gaseous dielectrics have been successfully developed recently to identify the replacement gases for SF_6_ [13,14,15]. It is believed that molecular structure plays a significant role in charge transport in dielectric liquids [16]. Knowledge of the chemical composition of insulating liquids is important to revealing the relationship between the compositions and the functional properties of the liquid [17]. However, the insulating oils usually consist of a variety of components mixed in specific proportions, whether they are part of paraffinic, iso-paraffinic, naphthenic, synthetic ester, or natural ester liquids. As a result, identifying an appropriate structure–activity relationship for the electrical performance of the pure compounds remains a challenge.

In this work, a variety of SAR models for the key properties of the compounds involved in dielectric liquids, including pulse breakdown strengths, AC breakdown voltages, dielectric constants, flash points, and kinematic viscosities, are proposed for the first time by means of first-principle electrostatic potential parameters and the group-additivity approach. It is worth noting that the present theoretical models are designed specifically only for the pure components involved in real insulating oils. The properties should be understood to be the inherent performance under identical actual operation conditions, such as antioxidant additives, nanofluids, and multiple stresses. More realistic models for real-time applications might be obtained by means of the procedures outlined in the present work with the extended experimental data. Rather than a prediction on the properties of complex oils subjected to various factors (e.g., oxidation, stressing, faults, etc.), the theoretical findings of the current work provide new insights into the predictive screening and rational design of the molecular structures of insulating fluid alternatives.

## 2. Results and Discussion

### 2.1. Electrical Strength

As dielectric insulating liquids, a high dielectric strength to withstand electrical stresses is the most important property for the operation and performance of a transformer. By carefully reviewing all the experimental work carried out [18,19,20,21,22], it appears that significant discrepancy exists among the experimental results reported by different investigators. The experimental electrical properties were obtained in liquids containing various kinds of unavoidable impurities, leading all electrical properties of dielectric liquids so far to be extrinsic rather than intrinsic [18,19]. Moreover, the influence of electrode surface conditions on breakdown strength has long been noticed [20]. In addition, the breakdown performance depends on the applied voltage duration, hydrostatic pressure, temperature, and so on. The lack of experimental results under identical conditions might hamper the development of a unified SAR model.

**Figure 1:**
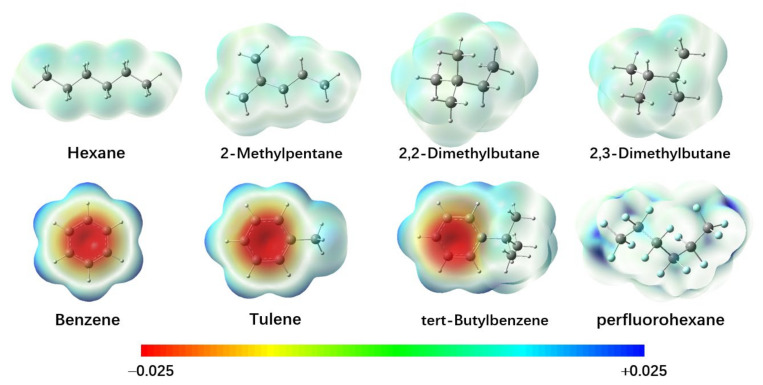
Contour plots for the surface electrostatic potential of the typical hydrocarbon liquids calculated at the M06-2X/6-31++G(d,p) level of theory.

**Figure 2:**
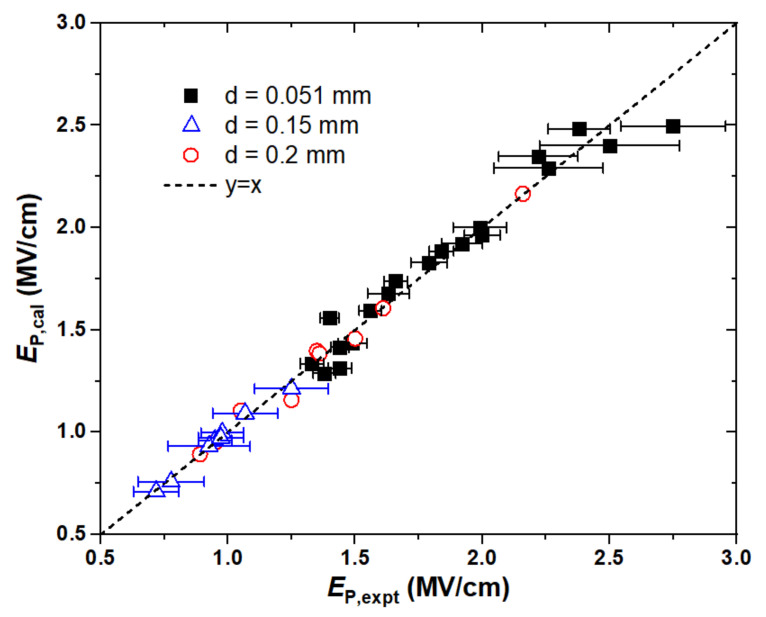
Correlations between experimental and predicted pulse electrical breakdown strengths for dielectric liquids with different gap lengths of the electrodes.

Two types of training datasets are employed in the present work. First is the pulse electric strength (*E*_P_). The pulse technique may reduce the effect of space charge so that the measured breakdown strengths are higher than those measured using DC/AC voltages. Three types of experimental data at ambient temperature and pressure were collected to serve as the training sets: (1) the electric strengths for 12 saturated (*n*-pentane, *n*-hexane, *n*-heptane, *n*-octane, *n*-nonane, *n*-decane, *n*-tetradecane, 2-methylpentane, 2,2-dimethylbutane, 2,3-dimethylbutane, 2,4-dimethylpentane, 2,2,4-trimethylpentane) and 7 aromatic (benzene, methylbenzene, ethylbenzene, *n*-propylbenzene, isopropylbenzene, *n*-butylbenzene, ter-butylbenzene) hydrocarbon liquids measured by applying a single pulse of voltage breakdown with the duration time of 1.65 microseconds to the liquid between hemispherical stainless-steel electrodes spaced 0.051 mm apart [18,19,20], (2) the electric strengths for 8 hydro- and fluorocarbon liquids (*n*-pentane, perfluoro-*n*-pentane, *n*-hexane, perfluoro-*n*-hexane, methylcyclohexane, perfluoro-methylcyclohexane, dekalin, perfluorodekalin) measured with electrodes spaced with a gap length of 0.15 mm in the uniform field [21], and (3) the electric strengths for 9 liquids (e.g., alkanes, benzenes, alcohols, CCl_4_) measured with electrodes spaced with a gap length of 0.2 mm and a pulse duration of 4.5 microseconds [22]. The experimental *E*_P_ data, with a total of 36 molecules, are listed in Table S1 in the Supplementary Materials. It should be noted that the pulse electric strength exhibits strong dependence on the gap lengths of the electrodes and the pulse duration. For example, the *E*_P_ values of *n*-hexane were measured to be 1.56 MV/cm, 0.978 MV/cm, and 1.35 MV/cm for the gap lengths of 0.051 mm, 0.15 mm, and 0.2 mm, respectively. Therefore, the independent model was developed for the individual gap distance. The coefficient of variation for the measurements ranged from 2 to 20%. The electrostatic potential surfaces for several molecules are shown in Figure 1. The best fit for electrical strengths between theory and experiment is illustrated in Figure 2.

For the hydrocarbons in mineral insulating oils, including straight- and branched chain C_n_H_2n+2_ (*n* = 5–14), cyclo-paraffins C_6_H_11_CH_3_ and decalin, and alkyl-benzenes, the experimental pulse electrical strengths could be reproduced well using the electrostatic parameters as follows:

*d* = 0.051 mm (*R*^2^ = 0.95, RMSE = 0.090):

(1) $$E_{P}=17.75{\left(A_{s}-1.02\right)}^{0.12}+0.0904\nu \sigma^{2}+0.00307\Pi -6.85{\left(\rho A_{s}^{+}/\Omega \right)}^{1/4}$$

*d* = 0.15 mm (*R*^2^ = 0.98, RMSE = 0.019):

(2) $$E_{P}=0.00188{\left(A_{s}+21.2\right)}^{5}+5211.1\nu \sigma^{2}+1.38E4\Pi -1.34E4\Omega -2.17E4V_{a}$$

*d* = 0.2 mm (*R*^2^ = 0.99, RMSE = 0.042):

(3) $$E_{P}=-3.32{\left(A_{s}-0.71\right)}^{3/4}-0.0296\nu \sigma^{2}+0.0365\Pi +0.126\rho A_{s}^{+}/\Omega$$

Apparently, the pulse breakdown strength is a function of the electrode gap length in addition to the molecular structures. For the smallest gap (0.051 mm) and the largest gap (0.2 mm) of our concern, the SAR models involve the same descriptors, namely, the total surface area *A*_s_, product of balance, and total variance νσ^2^, which represents the variability of the positive and negative surface potentials; the average deviation Π, which measures the internal charge separation or local polarity; and a product of ρ*A*_s_*^+^* divided by ovality, as indicative of the molecular topological characteristic. The dependence of *E*_P_ on the individual descriptor is fairly different, even though the training data for 0.051 mm and 0.2 mm sets covered similar molecular structures such as alkanes and benzenes. For instance, as *A*_s_ and νσ^2^ increase, *E*_P_ increases for d = 0.051 mm but decreases for d = 0.2 mm. The reverse observation exists for the composite descriptor $\rho A_{s}^{+}/\Omega$. The same correlation to Π for both 0.05 mm and 0.2 mm is obtained but the proportion is larger for the later. In the case of d = 0.15 mm, the liquids include fluorinated alkanes and dekalins. It appears that the best-fit correlation has to invoke another descriptor, i.e., *V*_a_, which is a measure of apparent value of the overall surface potentials. Moreover, the dependence of *E*_P_ on *A*_s_ is considerably enhanced by an order of 5. It implies that the fluorine-substitution might exert great influence on the pulse electrical strength of the mineral oils. For example, the perfluorinated C_5_F_12_, C_6_F_14_, C_6_F_11_CF_3_, and C_10_F_18_ molecules possess a higher *A*_s_ than the respective non-fluorinated compounds by about 25%, leading to roughly 0.2 MV/cm improvement on *E*_P_. Therefore, fluorination is capable of enhancing the pulse breakdown strength of the oils by about 20%.

All three models demonstrated that increasing Π can improve the pulse electrical strength. The higher the local polarity, the stronger the *E*_P_. As can be seen in Figure 1, aromatic compounds usually possess high internal charge separation on the electrostatic potential surface. This explains that the pulse electrical strengths of the aromatic hydrocarbons are higher than those of the saturated hydrocarbons. For instance, the *E*_P_ value of C_10_H_22_ is 1.92 MV/cm, while that of C_10_H_14_ (*n*-butyl-benzene) is as high as 2.75 MV/cm, as the value of Π increases from 2.2 kcal/mol to 6.0 kcal/mol. Meanwhile, the reduction in *E*_P_ due to the branched chains at the gap distance of 0.051 mm can be understood as well. The branched structures always lead to a smaller *A*_s_ and *A*_s_*^+^* than the straight chains, as indicative of the reduction in the cross-section for electron attachment. Although the total variance σ^2^, namely, the sum of the separate contribution from the positive and negative regions of the molecular surface, increases for the branched chains, the index parameter ν of the electrostatic balance is reduced. The more branched chain structures there are, the smaller the νσ^2^ value and the weaker the molecule’s capacity for attraction interactions. To compare *n*-C_6_H_14_ and the most-branched 2,2-dimethyl-butane, *A*_s_ and *A*_s_*^+^* decrease by 7% and 13%, respectively. The value of σ^2^ increases from 4.1 to 5.1 (kcal/mol)^2^, whereas the value of ν decreases from 0.16 to 0.10, leading to a reduction in νσ^2^ by about 18%. As a result, *E*_P_ decreases from 1.56 MV/cm to 1.33 MV/cm due to the branched structures. Note that the small increase in Π plays a minor role in view of its marginal contribution.

**Figure 3:**
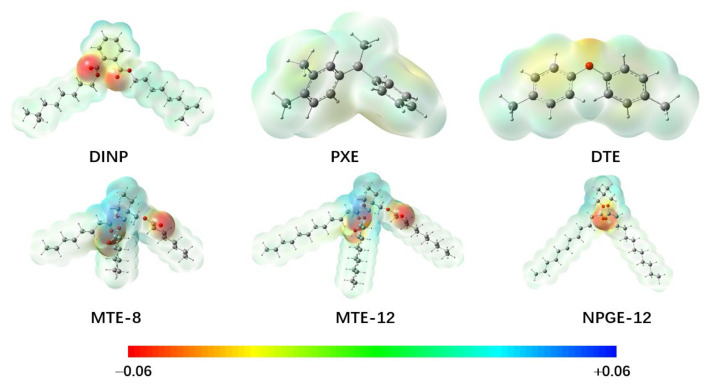
Contour plots for the surface electrostatic potential of the typical ester and aromatic liquids calculated at the M06-2X/6-31++G(d,p) level of theory.

**Figure 4:**
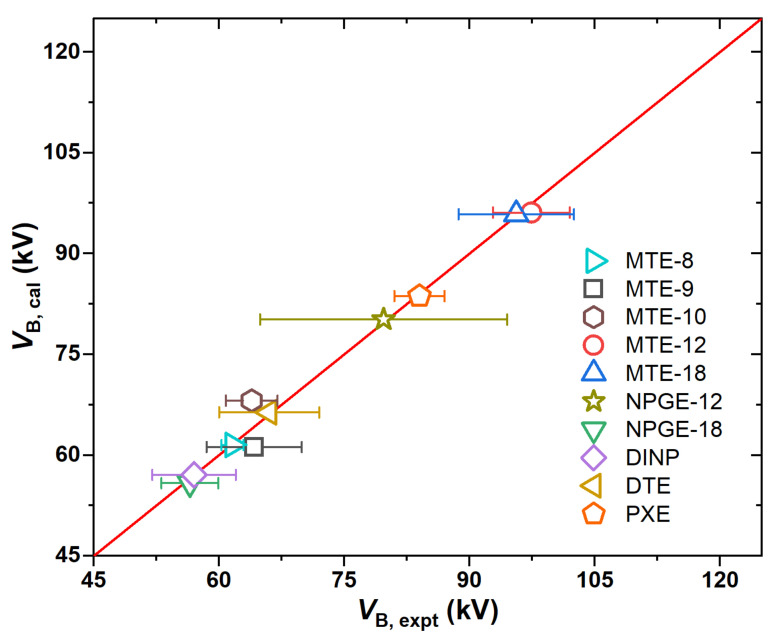
Correlations between experimental and predicted AC breakdown voltages for the ester dielectric liquids.

The second type of electrical property is the AC breakdown voltages (*V*_B_) for a total of 10 molecules that conform to the IEC 60156/61099 standard [23,24] (Table S2 in Supplementary Materials) [16,25,26]. The training dataset includes 5 trimethylolpropane ester insulating oils (MTE) containing 8–18 carbon atoms, two nepentyl glycol esters (NPGE) with 12 and 18 carbon atoms, and 3 aromatic compounds (DINP, PXE, and DTE), as shown partially in Figure 3. In comparison with the hydrocarbons in mineral oils, the esters exhibit different characteristics in the molecular electrostatic potentials, implying a different charge transport mechanism in the ester-based insulating oils from the paraffin-based oils. However, it is interesting to find that a similar SAR model to that of the paraffin-based oils could also be obtained for the ester-based oils, as shown in Figure 4:

(4) $$V_{B}=991.4A_{s}+0.756\nu \sigma^{2}+15.8\Pi -1300.6\Omega +590.9\sqrt[{3}]{V_{a}}-13.0W_{\mathrm{gt}}+1349.7$$

The predicted breakdown voltages are in good agreement with the experimental data, as indicated by *R*^2^ = 0.99 and RMSE = 1.71 kV. Some large error bars could be due to the impurities and moisture contents in the experimental fluids together with any random uncertainties. *V*_B_ shows a linear increase toward νσ^2^ and Π, which is the same as those in the *E*_P_ model. However, the contribution from the parameter Π, which is an average absolute deviation of the positive and negative potentials on the molecular surface, is enhanced significantly. Meanwhile, *V*_B_ shows a linear dependence on *A*_s_, indicating that the molecular size exerts a more significant effect on *V*_B_ than on *E*_P_. In addition, the global electrostatic parameter *V*_a_ has a positive contribution to *V*_B_, implying that the breakdown strength could be enhanced by increasing the net surface electrostatic potentials to capture electrons. For instance, the *V*_a_ value for MTE-12 (see Figure 3) is 2.01 au, while that for NPGE-12 is only 0.79 au, leading to a higher *V*_B_ for the former than the later by about 18 kV. The negative contribution to *V*_B_ is due to molecular ovality and weight. Such a topologic effect can be illustrated by MTE-12 and MET-18. As observed experimentally, the *V*_B_ of MET-12 is slightly higher than that of MET-18 because of the weaker ovality in shape and lower molecular weight, even though the *A*_s_ parameter for MET-12 is smaller by roughly 40% than that for MET-18. Therefore, the present SAR model provides insights into the rational design of ester-based oils by interplaying with the electrostatic and topologic parameters of the molecules.

It is worth comparing the correlations for insulating liquids with those for gases. On the basis of the 43 gaseous dielectrics, the following correlation for the dielectric strength with respect to SF_6_ was deduced, viz. [27,28]:

(5) $$E_{r}=0.30{\left(A_{s}+0.78\right)}^{2}+0.92\nu \sigma^{2}-1.84\Pi -0.039{\left(\rho A_{s}^{+}/\Omega \right)}^{2}$$

Evidently, the electrical strengths for both liquids and gases could be described well by the similar electrostatic potential parameters, being indicative of the inherent dependence of insulating property on the molecular structures at the microscopic level. However, some differences between liquids and gases do exist. While *E*_P_ and *V*_B_ of the liquids appear to be positively proportional to *A*_s_ and $\nu \sigma^{2}$ as for *E*_r_ of the gases, the dependence on Π is always opposite. Apparently, increasing of the local polarity can improve the electrical performance in the liquid phase but leads to deterioration in the gas phase.

### 2.2. Flash Point

The flash point per the ASTM D93 method is an important criterion for the fire and explosion hazards associated with transformers [29]. A total of 37 experimental closed-cup flash points were chosen in the present work to develop the SAR model [20,21,22,25,26]. All five descriptors and the experimental data are listed in Table S3. Both mineral and ester-based oils are included in the training set, with a wide range of the flash points from −48 °C (2,2-dimethylbutane, C_6_H_14_) to 310 °C (trimethylolpropane trioleate, C_60_H_110_O_6_). The optimized correlation is as follows:

(6) $$T_{f}=312.8{\left(A_{s}+0.94\right)}^{0.56}+0.070\sigma^{2}+3.32\Pi -20.9/\rho^{2}-0.71W_{\mathrm{gt}}$$

**Figure 5:**
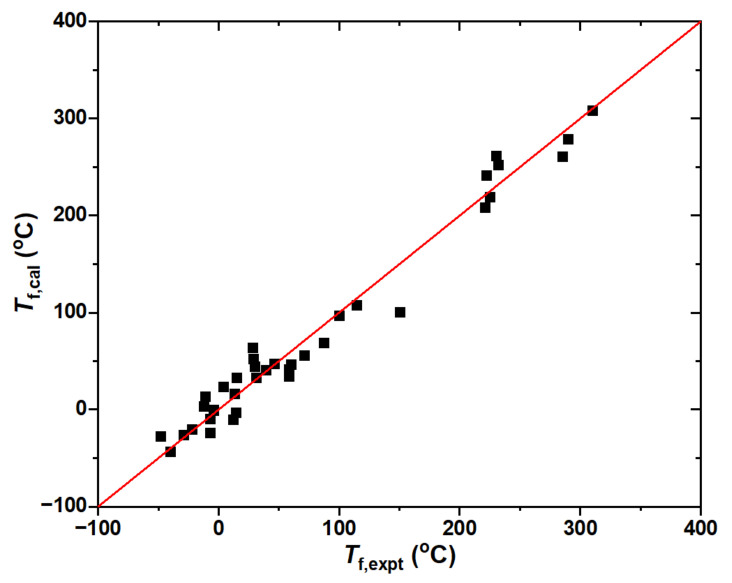
Correlations between experimental and predicted closed-cup flash points for the dielectric liquids.

The good agreement between the experiment and the theory is illustrated in Figure 5. The correlation coefficient *R*^2^ = 0.97 and the RMSE is about 18 °C. The united SAR model of the flash points for mineral and ester oils is very promising in view of the distinct electronic structures of hydrocarbons and esters.

It was assumed that the flash point is mainly a function of volatility and molecular weight. Increasing the molecular weight increases the flash point. However, as can be seen in the SAR model, *T*_f_ is indeed inversely proportional to the molecular weight. While the molecular densities of the oils are similar to each other in the range of 0.5~0.7 g/cm^3^, the flash point is determined mainly by three electrostatic potential parameters, in which the maximum contribution belongs to *A*_s_ together with σ^2^ and Π. It is worth noting that both σ^2^ and Π descriptors correspond to different determinants of the noncovalent interactions. While σ^2^ is an indicator of a molecule’s net electrostatic interactive tendencies, Π measures the internal charge separation or local polarity, although σ^2^ covers a much greater range of values than Π. Therefore, the intermolecular force governs the volatility of an insulating oil. As a result, the extremely high flash point for C_60_H_110_O_6_ is determined by its largest *A*_s_ (e.g., 12.8 nm^2^) and 146.1 (kcal/mol)^2^ in the training dataset.

### 2.3. Dielectric Constant

The dielectric constant, also known as the relative permittivity, represents the ability of insulating liquid to store electrical energy. Usually, it is related to the dipole moment and polarization characteristics of the molecule. Since mineral oils involve fewer polar molecular structures that contribute to their low dielectric constants, there is a significant difference between polyol esters and mineral oil in terms of dielectric constants due to their different polarity in nature.

**Figure 6:**
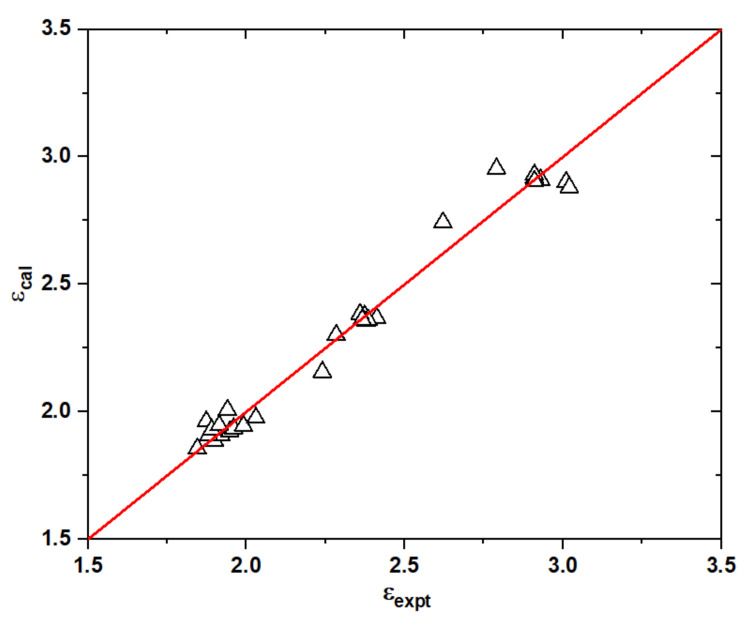
Correlations between experimental and predicted dielectric constants for various dielectric liquids.

A total of 33 liquids covering both mineral oils and esters were employed to develop the SAR model for the dielectric constants in a unified manner [20,21,22,25,26]. As shown in Figure 6 and Table S4, the dielectric constants are in the range of 1.8–3.0 and catalogued into three classes in the following order: esters > aromatic hydrocarbons > saturated hydrocarbons. The best-fit to the experimental data as achieved with the following expression, viz.,

(7) $$\varepsilon =2.79{\left(A_{s}-1.15\right)}^{0.028}-1.59\Pi^{-0.77}$$

Although only two descriptors were used, the correlation coefficient *R*^2^ is 0.98, with RMSE = 0.06. Therefore, the dielectric constants for the insulating oils could be deduced readily by the electrostatic parameters *A*_s_ and Π. Clearly, the dielectric constant is directly proportional to *A*_s_ and Π, but the dependence on the surface area is very weak. This explains the high but similar dielectric constants of esters of around 3.0. On the other hand, the parameter Π can be interpreted as the local polarity or internal charge separation that is presented even in molecules with a zero dipole moment. The values of Π for the aromatic compounds and esters are generally 6–9 kcal/mol, whereas those for saturated alkanes are around 2 kcal/mol. For example, benzene is a non-polar molecule, but it has a significant local polarity of Π = 8.2 kcal/mol, accounting for its dielectric constant of 2.28. Therefore, the local polarity is expected to be a determinant of the dielectric constant rather than the molecular dipole moment.

### 2.4. Kinematic Viscosity

The viscosity of the insulating liquid is important for many functional properties in electrical equipment. The liquid must be fluid enough to circulate for heat transfer where the viscosity has the biggest impact. Poor or no circulation might lead to hot spots and overheating. Reducing viscosity is the main reason why vegetable oils or fats are transesterified to biodiesel. Moreover, viscosity plays an important role in the lubrication of moving parts to minimize friction and wear. Kinematic viscosity (*η*) at 40 °C is the parameter required by biodiesel and petrodiesel standards. It has been observed that the viscosity of synthetic esters is proportional to their mean molecular weight [25]. However, the kinematic viscosity can be significantly influenced by structures such as chain length, position, number, and double bonds. Moreover, it was suggested that viscosity is somehow related to the energy required for the internal rotation of the molecular linkages.

**Figure 7:**
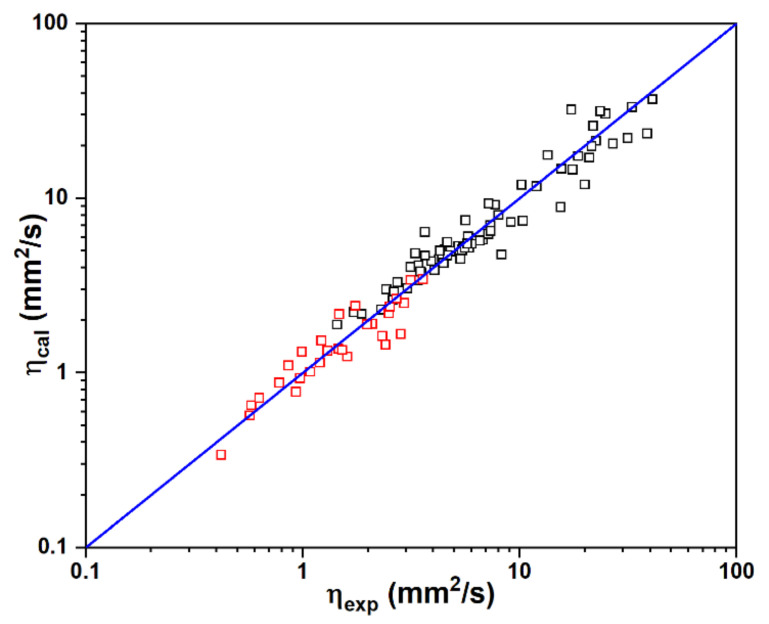
Correlations between experimental and predicted kinematic viscosities for 105 dielectric liquids. Red: 27 hydrocarbons, 1 ether and 1 ketone; black: fatty compounds (70 esters + 3 acids + 3 alcohols).

The experimental kinematic viscosities for 76 fatty compounds as well as 29 components of petrodiesel, as determined according to ASTM D445 [30], at 40 °C were employed for the purpose of model training (Table S5) [31]. The best-fit correlations between experimental and calculated kinematic viscosities for a total 105 compounds are shown in Figure 7 on the basis of five-parameter expression as follows:

(8) $$ln\eta =2.22lnW_{\mathrm{gt}}-2.59{\left(A_{s}-1.12\right)}^{0.30}+0.15{\left(\nu \sigma^{2}\right)}^{0.61}-7.85\sqrt[{15}]{\Pi }$$

The predicted kinematic viscosities are in good agreement with the experimental data with a value of RMSE 1.1 mm^2^/s. The correlation coefficient *R*^2^ is 0.953. It is evident that the kinematic viscosity is not only linearly proportional to the molecular weight but also is sensitive to the electrostatic characteristics in terms of the exponential relationship. In fact, it is unexpected that the kinematic viscosity shows a negative dependence on the molecular surface. The larger the *A*_s_, the lower the viscosity. Therefore, increasing the molecular size by the well-designed topological shape might be an appropriate approach to reducing the viscosity due to the large molecular weight. Interestingly, kinematic viscosity shows reverse dependence on $\nu \sigma^{2}$ and Π, which both measure the noncovalent interactions. A higher $\nu \sigma^{2}$ always leads to a higher viscosity because of the stronger intermolecular attraction. Meanwhile, viscosity could be reduced by increasing Π. As mentioned above, $\sigma^{2}$ and Π show very weak correlation despite what may appear to be an element of similarity between them. Although $\sigma^{2}$ refers to a molecule’s net electrostatic interactive tendencies, it does not provide any information about the degree of balance between positive and negative variances. The interactions can be strongly attractive only if both the positive and negative molecular surface potentials reach relatively large magnitudes; that is, ν should be near its maximum of 0.25. Therefore, the product $\nu \sigma^{2}$ that is related to how well a molecule interacts with others of its own kind is of key importance in the correlation. It is worth noting that the dependence of viscosity on Π is fairly weak, corresponding to a roughly constant interception. However, removing Π will considerably deteriorate the performance of the SAR model.

In order to gain insights into the structure dependence of the kinematic viscosities of the fatty compounds, the SAR models were attempted by means of group additivity. In contrast to the linear group contribution models, it was found that the kinematic viscosity cannot be characterized well unless the second-order relationship is applied, viz.:

(9) $$\eta =\underset{i}{\sum }{\left(n_{i}\eta_{i}\right)}^{2}$$

**Figure 8:**
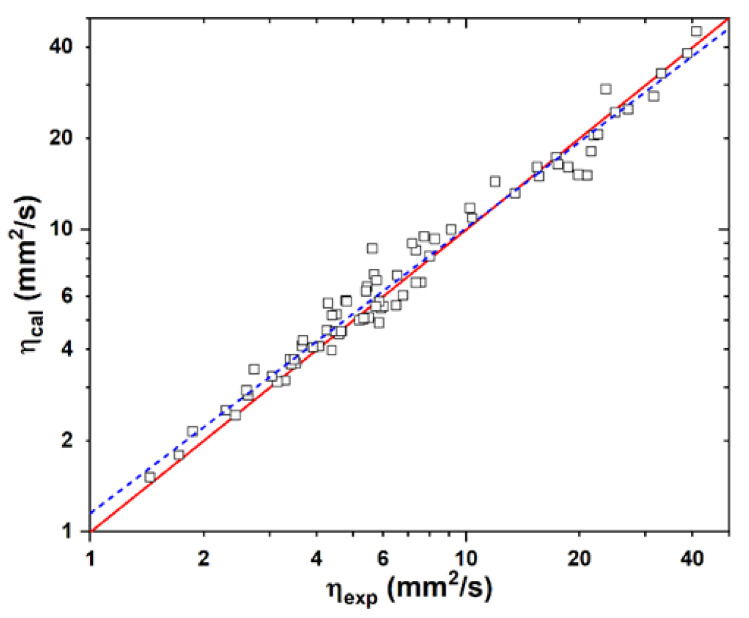
Comparison of the experimental kinematic viscosities at 40 °C with the group-addition calculated data for 76 fatty compounds. Solid line: y = x. Dashed line: y = 0.943x + 0.062.

The optimal group contributions are listed in Table 1. As shown in Figure 8, the experimental kinetic viscosities could be reproduced well for 76 fatty compounds (see Table S5) in terms of the designated seven structural units and three geometry factors. The correlation coefficient *R*^2^ is 0.967, with a value of RMSE = 1.6 mm^2^/s. For example, glyceryl trioleate has 1 CH, 44 CH_2_, 3 CH_3_, 3 CO_2_, 3 CC, 3 n_C_, and 7 m_C_, resulting in *η* = 1 × (−0.013) + 44 × 0.109 + 3 × 0.226 + 3 × (−0.0002) + 3 × (−0.0422) + 3 × 0.0145 + 7 × 0.0483 = 32.81 mm^2^/s, in comparison with the experimental value of 32.94 mm^2^/s.

It appears that the carbonyl group contributes negligibly to viscosity, so the monoester and polyol ester have similar viscosity. In contrast, the viscosity can be improved considerably by the OH group, as the intermolecular interaction is enhanced through hydrogen bonding. In addition, the chain length of the groups attached to the ester O site exerts a positive contribution to viscosity.

Although the unsaturated and saturated esters perform very differently in kinematic viscosity, the contribution due to the C=C double bond is not significant yet since the presence of one C=C bond only leads to a reduction in viscosity of 0.0422. Introduction of the trans C=C conformations will enhance the viscosity. Moreover, the position of the C=C bond might affect viscosity to some extent. The farther the C=C bond from the ester group, the higher the viscosity. For example, the viscosities of stearic acid ethyl ester (C_20_H_40_O_2_) can be lowered monotonically from 5.92 mm^2^/s to 4.78 mm^2^/s (oleic acid ethyl ester), 4.25 mm^2^/s (linoleic acid ethyl ester), and 3.42 mm^2^/s (linolenic acid ethyl ester) by introducing 1, 2, and 3 C=C double bonds in the cis-conformations, respectively. Dependence of the viscosity on the C=C bonds may originate from the intermolecular interactions due to the π-π stacking effect. Both numbers and positions of the C=C bonds of fatty acids could be effective targets for viscosity modification for the sake of molecular design.

In view of the carbon chains, the most significant contribution to kinematic viscosity results from the fully substituted C atoms, leading to an increase in viscosity of 0.49 mm^2^/s per C atom. In contrast, the less branched structures of CH and CH_2_ lead to a decrease and increase in viscosity of 0.013 and 0.109 mm^2^/s, respectively. The terminal CH_3_ group is capable of improving viscosity by 0.226 mm^2^/s. Therefore, the degree of methylation or the position of the CH_3_ group could be the key factor in the modification of esters to reduce viscosity. For example, the methylation of palmitic acid propyl ester (C_19_H_38_O_2_) to the terminal H atom to form the palmitic acid butyl ester results in an increase of 23% in the kinematic viscosity. In contrast, the formation of the palmitic acid iso-butyl ester only accounts for about a 13% increase in the kinematic viscosity. The group-additivity model provides a guidance for the systematic improvement on the kinematic viscosities of the synthetic esters.

## 3. Methods and Materials

Geometries of the molecules were fully optimized using the density functional M06-2X with the 6-31++G(d,p) basis set [32,33]. Harmonic frequencies were computed at the same level of theory to confirm that the geometry is a global minimum involving all the real frequencies. For the species with multiple conformations, the most stable conformer was located for further analysis. The electrostatic potential *V*_s_(*r*) on the molecular surface, i.e., the contour of electron density of *ρ*(*r*) = 0.001 au, was constructed by numerical grids according to the general interaction properties function [34]:

(10) $$V_{s}\left(r\right)=\underset{A}{\sum }\frac{Z_{A}}{\left|R_{A}-r\right|}-\int \frac{\rho \left(r^{\prime }\right)dr\prime }{\left|r^{\prime }-r\right|}$$

where *Z*_A_ is the charge on nucleus A, located at *R*_A_. Various statistical quantities were deduced on the basis of the surface potential, including:

(1) the total variance σ^2^:

(11) $$\sigma^{2}=\sigma_{+}^{2}+\sigma_{-}^{2}=\frac{1}{N}\overset{N}{\underset{i=1}{\sum }}{\left[V_{s}^{+}\left(r_{i}\right)-{\bar{V}}_{s}^{+}\right]}^{2}+\frac{1}{M}\overset{M}{\underset{j=1}{\sum }}{\left[V_{s}^{-}\left(r_{j}\right)-{\bar{V}}_{s}^{-}\right]}^{2}$$

where the average positive and negative potentials over the entire surface are

(12) $${\bar{V}}_{s}^{+}=\frac{1}{N}\overset{N}{\underset{i=1}{\sum }}V_{s}^{+}\left(r_{i}\right)$$

(13) $${\bar{V}}_{s}^{-}=\frac{1}{M}\overset{M}{\underset{j=1}{\sum }}V_{s}^{-}\left(r_{j}\right)$$

(2) the average deviation Π:

(14) $$\Pi =\frac{1}{N+M}\overset{N+M}{\underset{i=1}{\sum }}\left|V_{s}\left(r_{i}\right)-V_{a}\right|$$

where *V*_a_ is the overall average of *V*_s_(*r*),

(15) $$V_{a}=\left(N{\bar{V}}_{s}^{+}+M{\bar{V}}_{s}^{-}\right)/\left(N+M\right)$$

(3) the positive/negative balance parameter v:

(16) $$v=\frac{\sigma_{+}^{2}\sigma_{-}^{2}}{{\left(\sigma^{2}\right)}^{2}}$$

Moreover, the total surface area *A*_s_ is augmented by the positive surface area *A*_s_*^+^*, weight *W*_gt_, and density *ρ* = *W*_gt_/*V*, where *V* is the molecular volume. The molecular ovality Ω is calculated as follows:

(17) $$\Omega =\frac{A_{s}}{\left[4\pi {\left(3V/4\pi \right)}^{2/3}\right]}$$

All the ab initio calculations were carried out using the Gaussian16 programs. The quantitative optimizations of the correlating relationships to the experimental properties were obtained by the least-square fitting using the universal global optimization algorithm, as implemented in the 1stOpt statistical analysis program. Both correlation coefficients (*R*^2^) and the root mean square error (RMSE) were employed to assess the quality of the best-fit models, viz.:

(18) $$R^{2}=1-\frac{\sum_{i=1}^{n}{\left(y_{i}-Y_{i}\right)}^{2}}{\sum_{i=1}^{n}{\left(y_{i}-\bar{y}\right)}^{2}}$$

(19) $$RMSE=\sqrt{\frac{\sum_{i=1}^{n}{\left(y_{i}-Y_{i}\right)}^{2}}{n}}$$

where *y_i_* and *Y_i_* represents the experimental and model-predicted properties for the *i*th molecule involved in the training sets (Tables S1–S5), respectively. Various parameters and their combinations were attempted until the *R*^2^ values were greater than 0.95. It should be emphasized that our objective is not only to develop a predictive family-independent relationship but also gain insight into the nature of the physical interactions governing the properties of the oils. In order to not obscure the key factors that are involved, the properties are expressed in terms of the fewest possible computed descriptors that will permit a good representation of it. If the objective were simply the best possible correlations, more variables and wider variety of groups of molecules would be employed.

## 4. Conclusions

The structure–activity relationship is essential to designing new chemical compositions of insulating liquids with great improvements on the electrical performance. Systematic studies are carried out in the present work to develop the theoretical modes for pulse electrical strength, AC breakdown voltage, flash point, dielectric constant, and viscosity for various mineral and ester oils. It has been demonstrated that the surface electrostatic potential parameters are capable of giving good correlations between experimental and predicted properties in terms of the appropriate SAR models. The properties of any insulating liquids can be predicted efficiently based on the first-principle descriptors. More importantly, the novel molecules with the desired properties or overall performance can be designed rationally by playing with these common descriptors, i.e., *A*_s_, $\nu \sigma^{2}$, Π, and *W*_gt_, through structural and geometrical modifications.

## Figures and Tables

**Table 1:** Group contributions to the kinematic viscosities for fatty esters.

Descriptors	Description	*η* *_i_*
C	Group >C< with 4 single bonds	0.490
CH	Group >CH- with 3 single bonds	−0.013
CH_2_	Group -CH_2_- with 2 single bonds	0.109
CH_3_	Group CH_3_- with 1 single bond	0.226
CC	Group >C=C< double bond	−0.0422
CO_2_	-C(=O)O- carbonyl group	−0.0002
OH	Hydroxyl group	1.842
n_C_	Number of C atoms in the R group of -C(=O)O-R	0.0145
m_C_	Number of C atoms between -C=O/OH and C=C	0.0483
trans	Number of trans C=C conformations	0.113

## Data Availability

The original contributions presented in the study are included in the article material, further inquiries can be directed to the corresponding author.

## Supplementary Materials

The supporting information can be downloaded at: https://www.mdpi.com/article/10.3390/ijms25126654/s1.
