# Supplementary material for: Structure–Activity Relationship Models to Predict Properties of the Dielectric Fluids for Transformer Insulation System
Source: Int J Mol Sci. 2024 Jun 17;25(12):6654. doi: 10.3390/ijms25126654 (PMC11204222; doi:10.3390/ijms25126654)
Supplement: Supplementary file 1 [file ijms-25-06654-s001.zip › ijms-3038498-supplementary.pdf]

Supporting Information

Structure-Activity Relationship Models to Predict Properties of the Dielectric Fluids for Transformer Insulation System

Mi Zhang, Hua Hou, Baoshan Wang*
(College of Chemistry and Molecular Sciences, Wuhan University, Wuhan 430072, China)

*Corresponding author. E-mail: baoshan@whu.edu.cn

Table S1 Descriptors for the pulse electrical breakdown strengths model and the experimental data.

Molecules	CAS	A_s (nm^2)	ν	σ^2 (kcal/mol)^2	Π (kcal/mol)	ρ (A^{-3})	A_s^+ (Å^2)	Ω	V_a (kcal/mol)	E_p (MV/cm)
C_5H_{12}	109-66-0	1.48	0.145	4.23	2.28	0.306	105.78	1.147	1.93	1.44
C_6H_{14}	110-54-3	1.69	0.159	4.11	2.25	0.310	121.70	1.179	1.94	1.56
C_7H_{16}	142-82-5	1.90	0.166	4.03	2.23	0.314	138.18	1.211	1.95	1.66
C_8H_{18}	111-65-9	2.11	0.173	3.97	2.22	0.317	154.38	1.241	1.95	1.79
C_9H_{20}	111-84-2	2.32	0.179	3.92	2.21	0.319	170.60	1.271	1.95	1.84
$C_{10}H_{22}$	124-18-5	2.53	0.184	3.89	2.20	0.321	186.82	1.299	1.96	1.92
$C_{14}H_{30}$	629-59-4	3.38	0.198	3.79	2.17	0.325	251.56	1.404	1.93	2.00
C_6H_{14}	107-83-5	1.63	0.141	4.50	2.36	0.312	117.12	1.145	1.88	1.49
C_6H_{14}	75-83-2	1.56	0.104	5.10	2.64	0.315	106.33	1.099	2.08	1.33
C_6H_{14}	79-29-8	1.56	0.118	4.80	2.49	0.316	109.01	1.105	2.00	1.38
C_7H_{16}	108-08-7	1.74	0.129	4.60	2.45	0.320	123.39	1.125	2.01	1.44
C_8H_{18}	540-84-1	1.89	0.111	4.96	2.63	0.324	130.00	1.128	2.04	1.40
C_6H_6	71-43-2	1.26	0.233	42.56	8.22	0.355	70.95	1.084	1.22	1.63
$C_6H_5CH_3$	108-88-3	1.47	0.207	43.34	7.41	0.353	90.56	1.118	1.17	1.99
$C_6H_5CH_2CH_3$	100-41-4	1.67	0.200	45.67	6.74	0.351	105.41	1.145	1.15	2.26
$C_6H_5CH_2CH_2CH_3$	103-65-1	1.88	0.195	46.81	6.33	0.349	123.32	1.179	1.28	2.50
$C_6H_5CH(CH_3)_2$	98-82-8	1.83	0.205	46.94	6.11	0.351	115.36	1.156	1.19	2.38
$C_6H_5CH_2CH_2CH_2CH_3$	104-51-8	2.09	0.184	46.94	6.05	0.348	138.24	1.213	1.26	2.75
$C_{10}H_{14}$	98-06-6	1.95	0.200	47.87	5.88	0.354	126.77	1.147	1.29	2.22
C_5H_{12}	109-66-0	1.48	0.145	4.23	2.28	0.306	105.78	1.147	1.93	0.78
C_5F_{12}	678-26-2	1.84	0.017	16.31	2.80	0.732	125.97	1.158	2.25	0.95
C_6H_{14}	110-54-3	1.69	0.159	4.11	2.25	0.310	121.70	1.179	1.94	0.98
C_6F_{14}	355-42-0	2.06	0.011	15.07	2.69	0.739	143.06	1.173	2.19	1.25
$C_6H_{11}CH_3$	108-87-2	1.62	0.160	3.88	2.06	0.337	122.69	1.111	1.84	0.93
$C_6F_{11}CF_3$	355-02-2	2.07	0.007	14.21	2.59	0.746	149.70	1.158	2.13	1.07
$C_{10}H_{18}$	91-17-8	1.98	0.172	3.42	1.86	0.358	154.83	1.130	1.79	0.72
$C_{10}F_{18}$	306-94-5	2.50	0.007	11.73	2.36	0.759	201.37	1.175	2.10	0.97
CH_3OH	67-56-1	0.72	0.239	237.91	14.43	0.339	47.21	1.052	1.69	0.89
CH_3CH_2OH	64-17-5	0.94	0.222	213.77	11.40	0.338	69.06	1.076	2.16	0.95
C_6H_5Cl	108-90-7	1.45	0.202	44.09	9.02	0.416	69.38	1.114	1.64	1.05
C_6H_6	71-43-2	1.26	0.233	42.56	8.22	0.355	70.95	1.084	1.22	1.25
C_6H_{14}	110-54-3	1.69	0.159	4.11	2.25	0.310	121.70	1.179	1.94	1.35
$C_6H_5CH_3$	108-88-3	1.47	0.207	43.34	7.41	0.353	90.56	1.118	1.17	1.36
C_7H_{16}	142-82-5	1.90	0.166	4.03	2.23	0.314	138.18	1.211	1.95	1.50
$C_{10}H_{22}$	124-18-5	2.53	0.184	3.89	2.20	0.321	186.82	1.299	1.96	1.61
CCl_4	56-23-5	1.34	0.024	30.34	4.49	0.576	57.99	1.091	1.68	2.16

Table S2 Descriptors for the AC breakdown voltage model and the experimental data.

Molecules	CAS	A_s (nm^2)	ν	σ^2 $(kcal/mol)^2$	Π (kcal/mol)	Ω	V_a (kcal/mol)	W_{gt} (g/mol)	V_B (kV)
$C_{30}H_{56}O_6$	4826-87-3	6.71	0.155	226.81	8.68	1.675	2.07	512.76	61.6
$C_{33}H_{62}O_6$	126-57-8	7.34	0.154	218.54	8.11	1.727	2.03	554.84	64.2
$C_{36}H_{68}O_6$	78-17-1	7.97	0.152	211.97	7.67	1.775	2.02	596.92	63.9
$C_{42}H_{80}O_6$	25268-73-9	9.24	0.151	197.41	6.92	1.864	2.01	681.08	97.4
$C_{60}H_{110}O_6$	57675-44-2	12.79	0.165	146.07	6.30	2.077	1.76	927.51	95.6
$C_{29}H_{56}O_4$	10525-39-0	6.36	0.167	118.95	5.95	1.639	0.79	468.75	79.7
$C_{41}H_{76}O_4$	42222-50-4	8.73	0.167	97.54	5.62	1.821	0.85	633.04	56.5
$C_{26}H_{42}O_4$	28553-12-0	5.56	0.099	138.44	6.62	1.599	1.07	418.61	57.0
$C_{14}H_{14}O$	28299-41-4	2.60	0.212	44.87	8.11	1.274	0.07	198.26	66.0
$C_{16}H_{18}$	6196-95-8	2.78	0.186	46.65	7.29	1.260	0.06	210.31	84.0

Table S3 Descriptors for the closed-cup flash points model and the experimental data.

Molecules	CAS	A_s (nm^2)	σ^2 $(kcal/mol)^2$	Π (kcal/mol)	ρ (A^{-3})	W_{gt} (g/mol)	T_f (℃)
C_5H_{12}	109-66-0	1.48	4.23	2.28	0.306	72.15	-40.0
C_6H_{14}	110-54-3	1.69	4.11	2.25	0.310	86.18	-22.0
C_7H_{16}	142-82-5	1.90	4.03	2.23	0.314	100.20	-4.0
C_8H_{18}	111-65-9	2.11	3.97	2.22	0.317	114.23	13.0
C_9H_{20}	111-84-2	2.32	3.92	2.21	0.319	128.26	31.0
$C_{10}H_{22}$	124-18-5	2.53	3.89	2.20	0.321	142.28	46.0
$C_{14}H_{30}$	629-59-4	3.38	3.79	2.17	0.325	198.39	100.0
C_6H_{14}	107-83-5	1.63	4.50	2.36	0.312	86.18	-7.0
C_6H_{14}	75-83-2	1.56	5.10	2.64	0.315	86.18	-48.0
C_6H_{14}	79-29-8	1.56	4.80	2.49	0.316	86.18	-29.0
C_7H_{16}	108-08-7	1.74	4.60	2.45	0.320	100.20	-7.0
C_8H_{18}	540-84-1	1.89	4.96	2.63	0.324	114.23	-12.0
C_6H_6	71-43-2	1.26	42.56	8.22	0.355	78.11	-11.0
$C_6H_5CH_3$	108-88-3	1.47	43.34	7.41	0.353	92.14	4.0
$C_6H_5CH_2CH_3$	100-41-4	1.67	45.67	6.74	0.351	106.17	15.0
$C_6H_5CH_2CH_2CH_3$	103-65-1	1.88	46.81	6.33	0.349	120.19	30.0
$C_6H_5CH(CH_3)_2$	98-82-8	1.83	46.94	6.11	0.351	120.19	39.0
$C_6H_5CH_2CH_2CH_2CH_3$	104-51-8	2.09	46.94	6.05	0.348	134.22	71.0
$C_{10}H_{14}$	98-06-6	1.95	47.87	5.88	0.354	134.22	60.0
CH_3OH	67-56-1	0.72	237.91	14.43	0.339	32.04	12.0
CH_3CH_2OH	64-17-5	0.94	213.77	11.40	0.338	46.07	14.0
C_6H_5Cl	108-90-7	1.45	44.09	9.02	0.416	112.56	28.0
CCl_4	56-23-5	1.34	30.34	4.49	0.576	153.82	87.0
C_5F_{12}	678-26-2	1.84	16.31	2.80	0.732	288.03	29.0

**C_6F_{14}**	355-42-0	2.06	15.07	2.69	0.739	338.04	58.0
**$C_6H_{11}CH_3$**	108-87-2	1.62	3.88	2.06	0.337	98.19	-4.0
**$C_{10}H_{18}$**	91-17-8	1.98	3.42	1.86	0.358	138.25	58.0
**$C_{30}H_{56}O_6$**	4826-87-3	6.71	226.81	8.68	0.377	512.76	222.0
**$C_{33}H_{62}O_6$**	126-57-8	7.34	218.54	8.11	0.374	554.84	232.0
**$C_{36}H_{68}O_6$**	78-17-1	7.97	211.97	7.67	0.371	596.92	230.4
**$C_{42}H_{80}O_6$**	25268-73-9	9.24	197.41	6.92	0.366	681.08	290.0
**$C_{60}H_{110}O_6$**	57675-44-2	12.79	146.07	6.30	0.360	927.51	310.0
**$C_{29}H_{56}O_4$**	10525-39-0	6.36	118.95	5.95	0.364	468.75	225.0
**$C_{41}H_{76}O_4$**	42222-50-4	8.73	97.54	5.62	0.359	633.04	285.0
**$C_{26}H_{42}O_4$**	28553-12-0	5.56	138.44	6.62	0.377	418.61	221.0
**$C_{14}H_{14}O$**	28299-41-4	2.60	44.87	8.11	0.387	198.26	114.3
**$C_{16}H_{18}$**	6196-95-8	2.78	46.65	7.29	0.371	210.31	150.0

Table S4 Descriptors for the dielectric constants model and the experimental data.

**Molecules**	**CAS**	A_s **(nm²)**	**Π (kcal/mol)**	ε
**C_5H_{12}**	109-66-0	1.48	2.28	1.85
**C_6H_{14}**	110-54-3	1.69	2.25	1.90
**C_7H_{16}**	142-82-5	1.90	2.23	1.92
**C_8H_{18}**	111-65-9	2.11	2.22	1.95
**C_9H_{20}**	111-84-2	2.32	2.21	1.96
**$C_{10}H_{22}$**	124-18-5	2.53	2.20	1.99
**$C_{14}H_{30}$**	629-59-4	3.38	2.17	2.03
**C_6H_{14}**	107-83-5	1.63	2.36	1.88
**C_6H_{14}**	75-83-2	1.56	2.64	1.87
**C_6H_{14}**	79-29-8	1.56	2.49	1.89
**C_7H_{16}**	108-08-7	1.74	2.45	1.91
**C_8H_{18}**	540-84-1	1.89	2.63	1.94
**C_6H_6**	71-43-2	1.26	8.22	2.28
**$C_6H_5CH_3$**	108-88-3	1.47	7.41	2.38
**$C_6H_5CH_2CH_3$**	100-41-4	1.67	6.74	2.41
**$C_6H_5CH_2CH_2CH_3$**	103-65-1	1.88	6.33	2.37
**$C_6H_5CH(CH_3)_2$**	98-82-8	1.83	6.11	2.38
**$C_6H_5CH_2CH_2CH_2CH_3$**	104-51-8	2.09	6.05	2.36
**$C_{10}H_{14}$**	98-06-6	1.95	5.88	2.37
**CH_3OH**	67-56-1	0.72	14.43	32.70
**CH_3CH_2OH**	64-17-5	0.94	11.40	24.50
**C_6H_5Cl**	108-90-7	1.45	9.02	5.62
**CCl_4**	56-23-5	1.34	4.49	2.24
**$C_{30}H_{56}O_6$**	4826-87-3	6.71	8.68	3.01
**$C_{33}H_{62}O_6$**	126-57-8	7.34	8.11	2.93

**$C_{36}H_{68}O_6$**	78-17-1	7.97	7.67	2.91
**$C_{42}H_{80}O_6$**	25268-73-9	9.24	6.92	2.91
**$C_{60}H_{110}O_6$**	57675-44-2	12.79	6.30	2.79
**$C_{29}H_{56}O_4$**	10525-39-0	6.36	5.95	3.02
**$C_{41}H_{76}O_4$**	42222-50-4	8.73	5.62	2.91
**$C_{26}H_{42}O_4$**	28553-12-0	5.56	6.62	4.77
**$C_{14}H_{14}O$**	28299-41-4	2.60	8.11	3.66
**$C_{16}H_{18}$**	6196-95-8	2.78	7.29	2.62

Table S5 Descriptors for the kinematic viscosities model and the experimental data.

**Molecules**	**CAS**	**W_{gt} (g/mol)**	**A_s (nm^2)**	**ν**	**σ^2 (kcal/mol)^2**	**Π (kcal/mol)**	**η (mm^2/s)**
**$C_{57}H_{110}O_6$**	555-43-1	596.92	8.40	0.100	131.08	5.65	38.80
**$C_{57}H_{104}O_6$**	122-32-7	885.43	12.58	0.116	95.68	5.24	32.94
**$C_{57}H_{98}O_6$**	537-40-6	879.38	12.37	0.128	90.45	5.93	24.91
**$C_{57}H_{92}O_6$**	14465-68-0	873.34	11.74	0.143	85.26	6.71	17.29
**$C_{10}H_{20}O_2$**	334-48-5	172.26	2.65	0.204	168.64	6.82	5.63
**$C_{18}H_{34}O_2$**	112-80-1	282.46	4.25	0.202	114.82	5.68	19.91
**$C_{18}H_{32}O_2$**	60-33-3	280.45	3.66	0.215	122.90	7.01	13.46
**$C_{10}H_{22}O$**	112-30-1	158.28	2.63	0.184	151.68	5.35	8.24
**$C_{18}H_{36}O$**	143-28-2	268.48	3.69	0.222	94.05	5.22	17.53
**$C_{18}H_{34}O$**	506-43-4	266.46	3.70	0.194	106.96	5.86	11.94
**$C_{11}H_{22}O_2$**	110-42-9	186.29	2.87	0.084	140.55	6.16	1.72
**$C_{13}H_{26}O_2$**	111-82-0	214.34	3.29	0.083	133.00	5.63	2.43
**$C_{15}H_{30}O_2$**	124-10-7	242.40	3.69	0.126	93.94	4.94	3.30
**$C_{17}H_{34}O_2$**	112-39-0	270.45	4.13	0.083	118.31	4.91	4.38
**$C_{19}H_{38}O_2$**	112-61-8	298.50	4.56	0.084	111.66	4.65	5.85
**$C_{19}H_{36}O_2$**	112-62-9	296.49	4.47	0.097	97.68	5.33	4.51
**$C_{19}H_{34}O_2$**	112-63-0	294.47	3.99	0.114	91.48	6.26	3.65
**$C_{19}H_{32}O_2$**	301-00-8	292.46	4.33	0.098	97.91	6.60	3.14
**$C_{19}H_{36}O_3$**	141-24-2	312.49	4.53	0.145	126.29	6.93	15.44
**$C_{12}H_{24}O_2$**	110-38-3	200.32	3.09	0.067	141.20	5.85	1.87
**$C_{14}H_{28}O_2$**	106-33-2	228.37	3.52	0.068	133.24	5.41	2.63
**$C_{16}H_{32}O_2$**	124-06-1	256.42	3.94	0.068	125.63	5.05	3.52
**$C_{18}H_{36}O_2$**	628-97-7	284.48	4.36	0.069	118.82	4.77	4.57
**$C_{20}H_{40}O_2$**	111-61-5	312.53	4.78	0.069	112.59	4.53	5.92
**$C_{20}H_{38}O_2$**	111-62-6	310.51	4.70	0.081	97.62	5.19	4.78
**$C_{20}H_{36}O_2$**	544-35-4	308.50	4.62	0.087	96.69	5.86	4.25
**$C_{20}H_{34}O_2$**	1191-41-9	306.48	4.56	0.096	91.24	6.42	3.42
**$C_{13}H_{26}O_2$**	30673-60-0	214.34	3.31	0.058	140.66	5.62	2.30
**$C_{15}H_{30}O_2$**	3681-78-5	242.40	3.73	0.059	132.62	5.21	3.04
**$C_{17}H_{34}O_2$**	14303-70-9	270.45	4.15	0.060	125.33	4.90	4.05
**$C_{19}H_{38}O_2$**	2239-78-3	298.50	4.57	0.061	118.60	4.64	5.30

**$C_{21}H_{42}O_2$**	3634-92-2	326.56	4.99	0.062	112.37	4.42	6.78
**$C_{21}H_{40}O_2$**	111-59-1	324.54	4.91	0.073	97.46	5.07	5.44
**$C_{21}H_{38}O_2$**	38433-95-3	322.53	4.84	0.079	96.69	5.70	4.39
**$C_{17}H_{34}O_2$**	110-27-0	270.45	4.11	0.071	108.80	4.66	3.91
**$C_{19}H_{38}O_2$**	142-91-6	298.50	4.53	0.072	102.31	4.43	5.20
**$C_{14}H_{28}O_2$**	30673-36-0	228.37	3.52	0.056	140.29	5.39	2.60
**$C_{16}H_{32}O_2$**	106-18-3	256.42	3.94	0.057	132.19	5.04	3.39
**$C_{18}H_{36}O_2$**	110-36-1	284.48	4.36	0.058	125.23	4.76	4.47
**$C_{22}H_{40}O_2$**	2634-45-9	336.55	5.05	0.076	96.58	5.55	4.80
**$C_{16}H_{32}O_2$**	37811-72-6	256.42	3.90	0.062	126.57	4.88	3.48
**$C_{18}H_{36}O_2$**	25263-97-2	284.48	4.32	0.063	118.52	4.61	4.65
**$C_{20}H_{40}O_2$**	110-34-9	312.53	4.74	0.064	111.37	4.39	6.02
**$C_{15}H_{28}O_2$**	56219-06-8	240.38	3.63	0.097	107.47	6.07	2.73
**$C_{17}H_{32}O_2$**	1120-25-8	268.43	3.99	0.094	108.36	5.47	3.67
**$C_{19}H_{36}O_2$**	2777-58-4	296.49	4.47	0.109	93.23	5.21	4.64
**$C_{19}H_{36}O_2$**	14620-36-1	296.49	4.51	0.101	98.42	5.26	5.51
**$C_{19}H_{36}O_2$**	1937-62-8	296.49	4.51	0.103	94.36	5.22	5.86
**$C_{19}H_{36}O_2$**	1937-63-9	296.49	4.47	0.095	97.93	5.34	4.29
**$C_{19}H_{36}O_2$**	6198-58-9	296.49	4.51	0.099	95.27	5.24	5.41
**$C_{19}H_{34}O_2$**	2566-97-4	294.47	4.46	0.106	93.35	5.93	5.33
**$C_{21}H_{40}O_2$**	2390-09-2	324.54	4.89	0.095	93.01	5.05	5.77
**$C_{23}H_{44}O_2$**	1120-34-9	352.59	5.31	0.094	89.27	4.83	7.33
**$C_{10}H_{22}$**	124-18-5	142.28	2.53	0.184	3.88	2.19	0.97
**$C_{10}H_{22}O$**	693-65-2	158.28	2.67	0.047	90.54	4.19	1.08
**$C_{10}H_{20}O$**	693-54-9	156.27	2.55	0.103	195.62	6.91	1.47
**$C_{10}H_{20}O_2$**	1731-84-6	172.26	2.66	0.084	144.87	6.48	1.44
**C_6H_{14}**	110-54-3	86.18	1.69	0.157	4.10	2.25	0.42
**$C_{11}H_{24}$**	1120-21-4	156.31	2.74	0.188	3.85	2.19	1.20
**$C_{12}H_{26}$**	112-40-3	170.33	2.95	0.191	3.83	2.18	1.46
**$C_{14}H_{30}$**	629-59-4	198.39	3.38	0.197	3.78	2.17	2.09
**$C_{15}H_{32}$**	629-62-9	212.41	3.59	0.198	3.75	2.16	2.49
**$C_{16}H_{34}$**	544-76-3	226.44	3.80	0.201	3.75	2.15	2.93
**$C_{16}H_{34}$**	4390-4-9	226.44	3.14	0.101	5.01	2.71	3.14
**C_6H_6**	71-43-2	78.11	1.26	0.233	42.52	8.22	0.58
**C_7H_8**	108-88-3	92.14	1.47	0.207	43.33	7.41	0.57
**C_8H_{10}**	100-41-4	106.17	1.67	0.200	45.66	6.74	0.63
**C_9H_{12}**	103-65-1	120.19	1.88	0.195	46.80	6.33	0.78
**$C_{10}H_{12}$**	119-64-2	132.20	1.85	0.145	43.12	5.98	1.60
**C_6H_{12}**	110-82-7	84.16	1.44	0.115	3.39	1.90	0.93
**C_8H_{16}**	1678-91-7	112.21	1.81	0.143	3.72	1.98	0.86
**C_9H_{18}**	1678-92-8	126.24	2.02	0.172	3.86	2.00	0.99
**$C_{10}H_{20}$**	1678-93-9	140.27	2.23	0.171	3.77	2.01	1.22
**$C_{10}H_{18}$**	91-17-8	138.25	1.94	0.118	3.41	1.92	2.52
**$C_{10}H_{18}$**	493-01-6	138.25	1.98	0.173	3.42	1.86	1.75

**$CH_3(CH_2)_9CH=CH_2$**	112-41-4	168.32	2.89	0.163	24.56	3.39	1.30
**$CH_3(CH_2)_{11}CH=CH_2$**	1120-36-1	196.37	3.31	0.165	22.77	3.23	1.98
**$C_{16}H_{32}$**	629-73-2	224.43	3.70	0.205	15.95	2.96	2.70
**$C_{18}H_{36}$**	112-88-9	252.48	4.13	0.165	18.98	2.86	3.58
**$C_{11}H_{10}$**	90-12-0	142.20	1.91	0.235	42.10	8.24	2.41
**$C_{11}H_{10}$**	91-57-6	142.20	1.94	0.232	42.59	8.23	1.52
**$C_{12}H_{12}$**	573-98-8	156.22	2.08	0.213	43.27	7.89	2.83
**$C_{12}H_{12}$**	575-41-7	156.22	2.11	0.212	43.59	7.70	2.33
**$C_{20}H_{40}O_2$**	111-06-8	312.53	4.78	0.059	118.41	4.51	5.58
**$C_{22}H_{42}O_2$**	142-77-8	338.57	5.12	0.071	97.28	4.92	6.00
**$C_{22}H_{44}O_2$**	123-95-5	340.58	5.21	0.060	111.30	4.31	7.17
**$C_{20}H_{40}O_2$**	32153-86-9	312.53	4.72	0.064	101.19	4.35	5.73
**$C_{22}H_{42}O_2$**	142-77-8	338.57	5.06	0.073	90.21	4.81	6.56
**$C_{22}H_{44}O_2$**	55195-02-3	338.57	5.06	0.065	95.78	4.17	7.34
**$C_{24}H_{48}O_2$**	29806-73-3	368.64	5.50	0.060	102.13	4.01	7.98
**$C_{26}H_{52}O_2$**	22047-49-0	396.69	5.92	0.062	96.15	3.87	7.71
**$C_{26}H_{50}O_2$**	32953-65-4	394.67	5.97	0.068	91.74	4.53	9.11
**$C_{26}H_{50}O_2$**	19149-86-1	394.67	5.97	0.069	93.32	4.53	10.34
**$C_{21}H_{40}O_4$**	31335-74-7	356.54	5.00	0.076	157.51	5.99	7.18
**$C_{25}H_{48}O_4$**	27841-06-1	412.65	5.85	0.075	146.42	5.44	10.2
**$C_{29}H_{56}O_4$**	10525-39-0	468.75	6.69	0.076	135.69	5.03	15.58
**$C_{41}H_{76}O_4$**	42222-50-4	633.04	9.05	0.093	99.87	4.96	26.90
**$C_{37}H_{68}O_8$**	3008-50-2	640.93	8.62	0.083	150.18	6.15	21.84
**$C_{45}H_{84}O_8$**	13784-61-7	753.14	10.30	0.083	137.79	5.50	23.49
**$C_{30}H_{56}O_6$**	4826-87-3	512.76	6.96	0.074	153.42	6.08	20.96
**$C_{36}H_{68}O_6$**	78-17-1	596.92	8.23	0.074	141.59	5.49	22.45
**$C_{60}H_{110}O_6$**	57675-44-2	927.51	12.55	0.099	92.41	5.15	40.95
**$C_{40}H_{74}O_5$**	21209-30-3	635.01	9.12	0.104	100.67	5.44	21.45
**$C_{43}H_{80}O_4$**	78948-50-2	661.09	9.36	0.086	99.60	4.82	31.47
**$C_{38}H_{70}O_4$**	928-24-5	590.96	8.56	0.103	92.59	5.16	18.62
